# Induction of Resistance Against *Plutella xylostella* (L.) (Lep.: Plutellidae) by Jasmonic Acid and Mealy Cabbage Aphid Feeding in *Brassica napus* L.

**DOI:** 10.3389/fphys.2018.00859

**Published:** 2018-07-10

**Authors:** Gadir Nouri-Ganbalani, Ehsan Borzoui, Maryam Shahnavazi, Alireza Nouri

**Affiliations:** ^1^Department of Plant Protection, Faculty of Agriculture and Natural Resources, University of Mohaghegh Ardabili, Ardabil, Iran; ^2^Department of Oral and Maxillofacial Radiology, Faculty of Density, AJA University of Medical Sciences, Tehran, Iran; ^3^Institute of Higher Education of Sabalan Ardabil, Ardabil, Iran

**Keywords:** induced resistance, growth indices, nutritional indices, glucosinolate, trypsin inhibitors

## Abstract

The diamondback moth, *Plutella xylostella* (L.), has become the most destructive insect pest of cruciferous plants, such as *B. napus* throughout the world including Iran. In this study, the induction of resistance was activated in oilseed rape plants (*Brassica napus* L.) using foliar application of jasmonic acid (JA) and mealy cabbage aphid either individually or in combination against diamondback moth. Induced resistance by inducers significantly reduced the population growth parameters, as well as the survival rate of immature *P. xylostella*. Also, the nutritional indices of *P. xylostella* were studied to evaluate the potential impact of induced resistance on the insect feeding behavior. The values of the efficiency of conversion of ingested food, the efficiency of conversion of digested food, relative consumption rate, and relative growth rate of *P. xylostella* on JA-treated plants were significantly reduced compared to control. These are because glucosinolates and proteinase inhibitors are induced following treatment of plants. Also, we found a significantly higher glucose oxidase activity in the salivary gland extracts of larvae fed on JA treatment. These results express that JA and/or Aphid application induces systemic defenses in oilseed rape that have a negative effect on *P. xylostella* fitness. These findings develop our knowledge the effects of induced defenses on *P. xylostella*.

## Introduction

Oilseed rape (*Brassica napus* L.; Brassicaceae) is a major oilseed crop in Iran and throughout the world. It is most important vegetable oil source with an annual growth rate exceeds that of palm (FAO, 2011). Also, the seeds of *B. napus* are the important source of dietary protein for humans globally (Alizadeh et al., 2015). This plant is attacked by several insect pests in the field that often necessitate control measures by the growers to protect the crop (Alford et al., 2003).

In recent years, the diamondback moth, *Plutella xylostella* (L.) (Lep.: Plutellidae), has become the most destructive insect pest of cruciferous plants including the oilseed rape (Soufbaf et al., 2010; Furlong et al., 2013). The larvae feed on host plants’ leaves, causing substantial crop losses (Akandeh et al., 2016). Currently, the chemical control is the primary tactic used to control the diamondback moth. Since frequent application of insecticide is required per season to obtain satisfactory control; therefore, the pest has become resistant to most of the registered insecticides (Shelton et al., 1993). Also, pesticide use has had adverse effects on the human health and environment (Saldo and Szpyrka, 2009). To avoid such undesirable consequences, many scientists have focused their attention on the use of less hazardous practices and/or methods to protect the crops.

One of the most environmentally sound and economically feasible insect control methods is the use of resistant cultivars and/or reinforcement of plant defense system. Various defense traits of host plants affect the fitness of herbivore insects (Rask et al., 2000; Harvey et al., 2007; Mithöfer and Boland, 2012; Soufbaf et al., 2012). The plant chemicals may act as repellents, deterrents, antinutrients, and antidigestive compounds that interfere with the physiology of the herbivore and reduce its developmental and survival rate (Kessler and Baldwin, 2001; Franceschi et al., 2005; Karban, 2011; War et al., 2011; Lu et al., 2014; Nikooei et al., 2015).

The induction of plant defenses by insect feeding is regulated via multiple signaling cascades. In cruciferous plants, defense systems against herbivores are induced by the jasmonic acid (JA) and salicylic acid (SA) pathways (de Vos et al., 2005; Hopkins et al., 2009), which can be induced by synthetic JA (War et al., 2011) and by phloem-feeding insects (Zhu-Salzman et al., 2004). These signaling pathways cross talk and may act antagonistically or synergistically (Zarate et al., 2007; Koornneef and Pieterse, 2008). It is reported that the profile and concentration of glucosinolates and myrosinase in cruciferous plants have significant effects on the fitness of their insect pests (Martin and Müller, 2007; Verkerk et al., 2009; Angelino et al., 2015; Popova et al., 2017).

Synthetic JA is not directly toxic or inhibitory to the herbivores (Kagale et al., 2004); but, it induces toxic chemicals (alkaloids, phenolics, and terpenoids) in the plant organs that can limit the attack of herbivores. The induced defenses may augment the plant’s constitutive defenses against subsequent attackers (Howe and Schaller, 2008; Poelman et al., 2008). Sharma et al. (2009) reported the high amounts of polyphenols and tannins in resistant wild relatives of pigeonpea as compared to the cultivated pigeonpea and introduced these compounds as plant resistance factors to *Helicoverpa armigera* (Hübner) (Lep.: Noctuidae). Mouttet et al. (2011) assessed the influence of pre-infestation by a first attacker on the performance of a second attacker. They resulted that the induction of plant defense reactions lead to the production of secondary metabolites and in this way, the first attack enhances the plant’s ability to resist the second attacker and reduction in its performance.

The individual effects of JA and herbivory in the induction of resistant in plants and their effect on subsequent herbivory have been well documented. Despite this abundance of researches, there exists a lack of studies to characterize the effects of JA and prior herbivory induced resistance in oilseed rape plants on the diamondback moth. Therefore, the aim of this research has been to study the induction of resistance on oilseed rape plants treated with JA and mealy cabbage aphid either individually or in combination against diamondback moth through the assessment of life table parameters, growth indices (GI), nutritional indices, and glucose oxidase activity (GOX) of the pest. This study provides an insight into the mode of action of JA- and herbivory-dependent defenses against subsequent pest and hence can help to find a solution to pest control management.

## Materials and Methods

### Chemicals

Substrates, synthetic JA, *o*-dianisidine, proteinase K, D-glucose, trypsin enzyme, and trichloroacetic acid (TCA) were purchased from Sigma Chemical Co. (St. Louis, MO). Horseradish peroxidase was purchased from Roche Co. (Grenzach-Wyhlen, Germany) and Tris, acetone, and potassium phosphate was purchased from Merck Co. (Darmstadt, Germany).

### Plant

Seeds of *B. napus* cultivar RGS_003_, as a semi-resistance cultivar, were obtained from the Plant and Seed Improvement Research Institute (Karaj, Iran). Plants were grown in 10 L plastic pots filled with a mixture of autoclaved soil (with the ratio of 2:1:1 field soil, sand, and vermicompost, respectively) and protected by 100-mesh muslin to prevent insect infestation. The pots were arranged in a randomized block design within the research greenhouse of the University of Mohaghegh Ardabili (Ardabil, Iran), set at 25°C with a natural photoperiod. Thirty-five-days-old plants were used for the experiments.

### Insects

A colony of *P. xylostella* used in the experiments was obtained from a cabbage field in Karaj (Iran), in September 2015. The colony has been maintained for about 15 months in the Laboratory of Entomology, University of Mohaghegh Ardabili, Ardabil, Iran, without any exposure to insecticide. They were reared on oilseed rape leaves inside a growth chamber that was set at 25°C, 60% relative humidity, and a photoperiod of 16:8 (L:D).

A colony of *Brevicoryne brassicae* L. (Hem.: Aphididae) used in the experiment was originally obtained from kohlrabi fields in Ardabil (Iran), in August 2016. The insects have been maintained for more than 4 months in the greenhouse without any exposure to insecticide. To maintain the aphid colony every 2 weeks, 10–15 apterous aphids were transferred from infected plants to healthy plants.

### Experiments

All following experiments including induction of resistance, life table parameters, GI, and nutritional indices were carried out under laboratory conditions inside a growth chamber, that was set at 25°C, 60% relative humidity, and a photoperiod of 16:8 (L:D).

### Jasmonic Acid Preparation

Synthetic JA was dissolved in acetone at a rate of 1 g ml^−1^ and dispersed in an appropriate volume of water to achieve 5 mM JA solution (Thaler et al., 1999). The control solution consisted of only acetone dissolved in water.

### Treatments Application

Resistance was artificially induced on oilseed rape by the foliar application of JA (5 mM), Aphid (*B. brassicae*), and JA plus Aphid.

In order to determine the induced resistant by JA, 30 oilseed rape plants were sprayed with JA and control solutions (15 plants for each solution). Total of 15 ml of solution was applied to each plant by a hand pressure sprayer. Plants treated with JA and control solutions were separated by using different cages to prevent volatiles induced by JA from eliciting defenses in control plants. After 2 days, the surface of plants was cleaned with water. Then, the treated and control plants were used for the bioassays or extractions.

In order to determine the induced resistant by *B. brassicae*, apterous adults (within 24 h) from the stock colony were transferred on central leaves of 15 caged oilseed rape plants at the rate of 20 females per plant. Fifteen plants were kept as a control without the prior release of aphids before *P. xylostella*. Aphids were removed from plants after being allowed to feed for 2 days. Then, the treated and control plants were used for the bioassay or extraction. During infestation with aphid, offspring were removed from the plants on a 12 h basis.

In order to determine the induced resistant JA plus Aphid, 15 plants were sprayed with JA solution. After that, *B. brassicae* adults (within 24 h) from the stock colony were transferred on central leaves of 15 caged oilseed rape plants at the rate of 20 females per plant. Treated and control plants were separated by using different cages to prevent volatiles induced by JA and Aphid from eliciting defenses in control plants. After 2 days, Aphids were removed from plants and the surface of plants was cleaned with water. Then, the treated and control plants were used for the bioassays or extractions.

### Life Table Parameters

To obtain *P. xylostella* eggs of the same age, 20 male–female pairs of the newly emerged moths from the stock colony were transferred to oviposition plastic cages (diameter 30 cm, depth 30 cm) covered with 100-mesh screen net for ventilation. Male and female adults were distinguished based on their abdomen; the tip of the abdomen in female moths is slightly swollen and in male moths is slender and elongated. After 12 h, laid eggs were collected and placed in the incubator until hatching. Once hatching, 60 *P. xylostella* newly hatched larvae (within 12 h) were released at the rate of 6 larvae per plant on 10 caged oilseed rape plants. Larvae were also released on caged oilseed rape plants that were not treated by JA and/or aphid, to be kept as controls. The plants were monitored daily until the immature stages of *P. xylostella* completed their development or died.

After eclusion, a pair of newly emerged adults (one male and one female) was transferred to plastic oviposition containers (diameter 11 cm, depth 12 cm); the containers were closed at the top with a 100-mesh screen net for ventilation. The number of pairs of tested moths for each host plant depended on their survival from the previous stage and ranged from 18 to 44 couples. A small cotton wick soaked in 10% honey solution was placed in each oviposition container to supply a source of carbohydrate to the adult feeding insects. Leaves from treated and control plants were replaced with fresh leaves every day, and the number of deposited eggs was recorded until the female’s death. The eggs were maintained for 10 days to estimate the percentage of hatched eggs (fertility).

The development time, immature survival rate, and fecundity were used to the calculation of the life table parameters. Calculations were made for age-stage survival rate (*s*_xj_) of *P. xylostella* on different treatments based on the method of Chi and Su (2006). Estimates for net reproductive rate (*R*_0_), the intrinsic rate of increase (*r*), finite rate of increase (*λ*), and mean generation time (*T*) of *P. xylostella* on different treatments were calculated based on Huang and Chi (2013).

### Ovipositional Preference Experiment

In order to determine the ovipositional preference of *P. xylostella* in a free-choice situation, the control, and treated plants were arranged in a randomized complete block design with five replications inside a metal cage (length 200 cm, width 180 cm, height 120 cm) covered by 100-mesh muslin screen net. Ten pairs of 24- to 48-h-old moths were randomly collected from the rearing chamber, released inside each cage, and provided a 10% sterile honey solution for adult feeding. The number of deposited eggs by females on each plant was counted and recorded 24 h after moth releasing.

### Growth Indices

Growth indices of *P. xylostella* were determined as described by Itoyama et al. (1999). To estimate the GI, we used growth and survival data from life table parameters experiment. Also, pupae obtained from the life table parameters experiment were individually weighed 24 h after pupation. In this study, GI of *P. xylostella* fed on control and treated plans were calculated using the following formulae:

Immature GI=(immature survival rate)/(immature duration)

Standardized insect−growth index (SII)=(pupal wt.)/(larval period)

Fitness index (FI)=(percentage of pupation×pupal wt.)/(immature duration)

where immature refer to larvae and pupae stages.

### Nutritional Indices

Nutritional indices were determined as described by Waldbauer (1968), Manuwoto and Scriber (1982), and Farrar et al. (1989) with some modifications by Dastranj et al. (2017). Initially, neonates were reared on the treated and control plants until the unset of fourth instar. This experiment carried out with seven replications (40 larvae in each) for each treatment. Nutritional indices were determined using the fourth instar larvae. Seven groups of 10 larvae each were prepared, weighted, and transferred into glass Petri dishes (12 cm diameter and 1.5 cm depth) containing the fresh oilseed rape leaf of each treatment or control. The petioles of the leaves were inserted into cotton ball soaked in water to maintain freshness. For 4 days, the initial fresh food, food remnant, and feces remaining at the end of each experiment were weighed daily. Also, the larvae in each Petri dish were checked daily for mortality or ecdysis. To establish the percentage of dry weight of the food, larvae, and feces, 20 specimens for each were weighed, oven-dried (48 h at 60°C), and subsequently re-weighed. Nutritional indices were calculated using the following formulas, based on dry weights:

$$\begin{array}{c} \text{Efficiency of conversion of ingested food }(\text{ECI})\\ =[(\text{insect wt. gain})/(\text{wt. food eaten})]\times 100 \end{array}$$

$$\begin{array}{c} \text{Efficiency of conversion of digested food }(\text{ECD})=\\ {}[(\text{insect wt. gain})/(\text{wt. food eaten}-\text{wt. frass})]\times 100. \end{array}$$

$$\begin{array}{c} \text{Relative consumption rate }(\text{RCR})=(\text{wt. food eaten})/\\ \left(\text{insect wt. at beginning of trial})\;(\text{time}\right) \end{array}$$

$$\begin{array}{c} \text{Relative growth rate }(\text{RGR})=(\text{insect wt. gain})/\\ \left(\text{insect wt. at beginning of trial})\;(\text{time}\right) \end{array}$$

### Preparation of Extract From the Whole Body of Larvae

To investigate GOX, neonates were reared on the treated and control plants until the beginning of fourth instar. In fourth instar, groups of 15 larvae (24–48 h old) were pooled in a precooled Teflon pestle and immediately homogenized in Nathathan’s saline (Christensen et al., 1991), containing proteinase inhibitor (PI) to inhibit digestive proteases in the saliva and cellular proteases released during homogenization, and used in whole body GOX experiments. The homogenate was centrifuged at 12,000 *g* for 15 min (4°C). The supernatant was used as the salivary gland extract.

### Glucose Oxidase Activity

Glucose oxidase activity was determined by the method of Kelley and Reddy (1988), with slight modification. To assay the GOX, the reaction mixture containing 0.17 mM *o*-dianisidine-HCl in potassium phosphate buffer (0.1 M; pH 7.0), 95 mM D-glucose, and 60 U ml^−1^ horseradish peroxidase was incubated at 37°C and saturated with oxygen. Then, 100 μl salivary gland extracts were added and, over 15 min, the change in absorbance at 460 nm min^−1^ was calculated to obtain the slope of the linear portion. For the control, 100 μl potassium phosphate buffer (0.1 M, pH 7.0) was added instead of the salivary gland extract. This experiment was repeated five times for each treatment and control.

### Extraction of Oilseed Rape Leaves

Initially, each extract of the leaves was prepared using liquid nitrogen to homogenize the whole leaf in a Tris–HCl buffer (50 mM; pH 8). A volume (1 ml) of homogenate was removed and placed in a 1.7-ml centrifuge tube. The tubes were vortexed and centrifuged at 8,000 *g* for 10 min at 4°C. The supernatant was taken and used as a source of inhibitor for the inhibition assays.

### Inhibitory Assay of Oilseed Rape Leaf Extract

The ability of oilseed rape leaf extract inhibitors to inhibit trypsin activity was determined by incubating a mixture of 40 μl trypsin (1 mg trypsin/10 ml 1 mM HCl), 100 μl of supernatant of leaf extract of plants containing trypsin inhibitors, and 160 μl Tris–HCl buffer (pH 8) for 30 min at 37°C. A control containing no enzyme extract with buffer was run simultaneously with the reaction mixture. Ten additional tubes (five without enzyme blanks, and five with enzyme but without leaf extract) were also prepared to determine the maximum enzyme activity. The reaction was terminated by adding 200 μl of TCA (10% w/v H_2_O), continued by cooling at 4°C for 30 min and centrifuging at 15,000 *g* for 10 min (4°C). One hundred microliters of supernatant were added to 100 μl of 2 M NaOH and the absorbance was read at 450 nm. This experiment was replicated five times.

### Glucosinolate Analysis

The collected leaves that originated from 24 plants (6 plants for each control and treatment) were freeze-dried and ground to a fine powder. Fifty grams of ground leaf material per sample was dissolved in methanol. The extract was analyzed as desulpho derivatives using the HPLC method described by van Dam et al. (2004). Results are expressed in micromoles of glucosinolate per gram dry mass.

### Data Analysis

All data calculated for each individual were subjected to the bootstrap method with 500 resampling for estimating the means, variances, and standard errors of population parameters. Difference between treatments was then compared by using the paired bootstrap test (Efron and Tibshirani, 1993; Akköprü et al., 2015). One-way ANOVA was used to compare the effects of the induced resistant on the GI, nutritional indices, and GOX activity of *P. xylostella* fed on control and treated plants and also enzyme inhibition by plant extracts. Means were compared at the *P* < 0.05 and Tukey’s HSD method using SAS 9.2 software (PROC GLM; SAS Institute, 2009). Correlation analysis of the life table parameters, GI, nutritional indices, and GOX activity of *P. xylostella* fed on control and treated plans with glucosinolate level of plants and leaf extract inhibitors was performed using SPSS 16.0.

## Results

Controls were compared in the experiments and, because there was no significant difference, mean of data from controls were presented in the tables and figures.

### Individual and Combined Effect of JA and Aphid on Life Table Parameters

**FIGURE 1:**
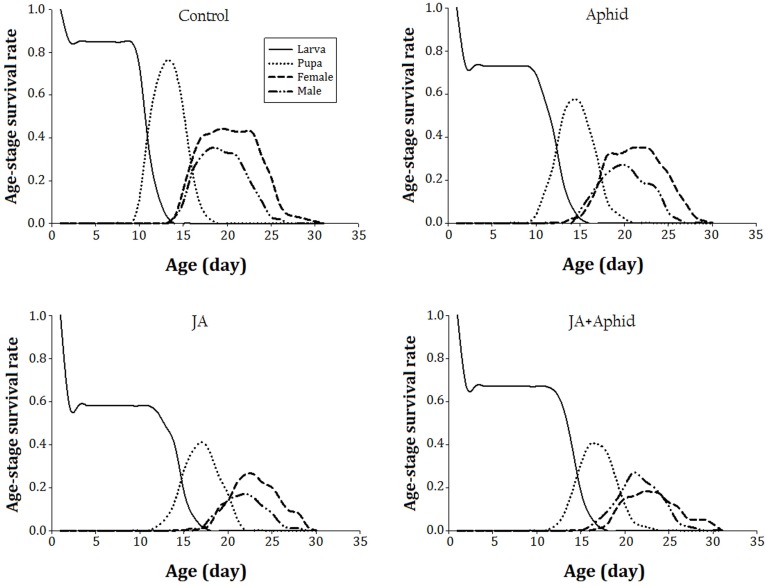
Age-stagesurvival rate (*s*_xj_) of *Plutella xylostella* fed on oilseed rape plants treated with jasmonic acid and/or aphid.

The age-stage specific survival rate (*s*_xj_) of *P. xylostella* on control and treated plants indicates the probability that a newborn survival to age *x* and develop to stage *j* (**Figure 1**). The immature developmental time was longer and the survival rate was lower on JA-treated plants. The survival rate of immature stages was approximate to 80% on the control plants. The survival rate of immature stages on JA-treated plants was 40% lower than those reared on the control plants.

The population growth parameters of *P. xylostella* fed on control and treated plants are presented in **Table 1**. The population fed on control plants had a higher net reproductive rate (*R*_0_; 63.73) and those reared on JA treatment had lowest *R*_0_ value (16.35). The difference in the intrinsic rate of increase (*r*) was statistically significant in control and *P. xylostella* fed on treated plants. The population fed on control plants had a much higher *r* value (0.1831 d^−1^) than those on JA-treated plants (0.1070 d^−1^). The finite rate of increase (*λ*) for *P. xylostella* populations varied from 1.1130 d^−1^ on JA-treated plants to 1.2010 d^−1^ on control plants. The mean generation time (*T*) was also different in control and *P. xylostella* fed on treated plants with the control plants promoting the fastest generation times (23.82 d).

**Table 1:** Life table parameters of *Plutella xylostella* fed on oilseed rape plants treated with jasmonic acid and/or aphid^1^.

Treatment	*R*_0_ (egg/female)	*r* (day^−1^)	*λ* (day^−1^)	*T* (day)
Control	63.73 ± 7.26^a^	0.1831 ± 0.0053^a^	1.2010 ± 0.0064^a^	23.82 ± 0.50^b^
Aphid	39.80 ± 5.21^b^	0.1542 ± 0.0056^b^	1.1668 ± 0.0066^b^	22.64 ± 0.32^c^
JA	16.35 ± 3.77^d^	0.1070 ± 0.0069^d^	1.1130 ± 0.0077^d^	25.91 ± 0.18^a^
JA + Aphid	22.67 ± 3.36^c^	0.1201 ± 0.0084^c^	1.1276 ± 0.0093^c^	25.86 ± 0.28^a^
*P*	<0.01	<0.01	<0.01	<0.01

*^1^The plants were treated with jasmonic acid (5 mM for 2 days) and/or aphid (20 adults for 2 days) prior to determining nutritional indices of P. xylostella. Means in a row followed by different letters are significantly different at P < 0.05 by using paired bootstrap test. R_0_, net reproductive rate; r, intrinsic rate of increase; λ, finite rate of increase; T, mean generation time. JA, jasmonic acid.*

### Individual and Combined Effect of JA and Aphid on Ovipositional Preference

**FIGURE 2:**
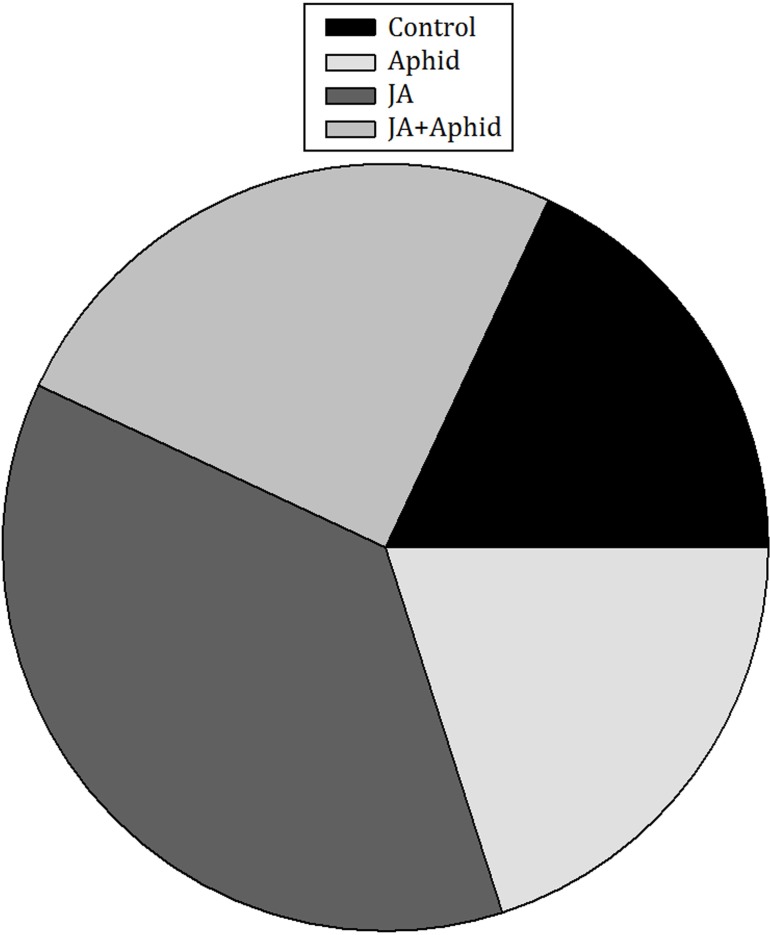
Ovipositional preference of *Plutella xylostella* on oilseed rape plants treated with jasmonic acid and/or aphid.in the free-choice situation. This experiment was replicated five times.

The number of deposited eggs by *P. xylostella* females significantly differed among control and treatments (*F*_3,16_ = 5.60; *P* = 0.0081). In 24 h, the numbers of deposited eggs on JA-treated plants exceeded other treatments and control (**Figure 2**). Females laid 2.0-, 1.5-, and 1.8-fold more eggs on JA-treated plants than on control, Aphid-treated plants, and JA + Aphid-treated plants, respectively.

### Individual and Combined Effect of JA and Aphid on Growth Indices

*Plutella xylostella* that fed on oilseed rape plants treated with JA, Aphid, or JA + Aphid showed statistically significant reductions in the immature GI, SII, and FI in comparison to those insects that fed on control plants (**Table 2**). The insects fed on control had the highest immature growth index (0.0528), while the lowest was on JA + Aphid treatment (0.0214). The SII of *P. xylostella* was 0.634 mg d^−1^ on control and 0.279 mg d^−1^ on JA treatment. The mean FI was 10.1, 10.5, and 21.8 mg d^−1^ on plants treated with JA, JA + Aphid, and Aphid, respectively. The control *P. xylostella* FI was 34.8 mg d^−1^, a value which was significantly higher than the FI of insects on treated plants.

**Table 2:** Growth indices of immature stage of *Plutella xylostella* fed on oilseed rape plants treated with jasmonic acid and/or aphid^1^.

Treatment	*N*	GI	SII (m/day)	FI (mg/day)
Control	79	0.0528 ± 0.0004^a^	0.634 ± 0.007^a^	34.8 ± 0.3^a^
Aphid	65	0.0404 ± 0.0005^b^	0.472 ± 0.007^b^	21.8 ± 0.3^b^
JA	48	0.0262 ± 0.0003^c^	0.279 ± 0.004^d^	10.1 ± 0.1^c^
JA + Aphid	43	0.0214 ± 0.0003^d^	0.312 ± 0.006^c^	10.5 ± 0.2^c^
df		3, 231	3, 231	3, 231
*F*		94.35	61.61	196.59
*P*		<0.01	<0.01	<0.01

*^1^The plants were treated with jasmonic acid (5 mM for 2 days) and/or aphid (20 adults for 2 days) prior to determining nutritional indices of P. xylostella. Mean values followed by different letters in the same column are significantly different (Tukey’s test, P < 0.05). GI, immature growth indices; SII, standardized insect-growth index; FI, fitness index; JA, jasmonic acid.*

### Individual and Combined Effect of JA and Aphid on Nutritional Indices

In general, the nutritional indices (ECI, ECD, RCR, and RGR) of fourth instar *P. xylostella* that fed on oilseed rape plants treated with JA, Aphid, or JA + Aphid were significantly lower than those fed on control plants (**Table 3**). The ECI of larvae varied from 3.49 to 4.45%, with the minimum on JA treatment and the maximum on control. Similarly, the ECD was the highest in control (5.77%) and the lowest on JA treatment (4.30%). The data revealed that the highest RCR was recorded for larvae fed on control plants (2.83 mg mg^−1^ d^−1^), while the lowest was on JA-treated plants (1.88 mg mg^−1^ d^−1^). The RGR was highest for larvae fed on control plants (0.126 mg mg^−1^ d^−1^), at an intermediate level on JA + Aphid-treated plants (0.091 mg mg^−1^ d^−1^), and the lowest on JA-treated plants (0.066 mg mg^−1^ d^−1^).

**Table 3:** Nutritional indices of fourth instar larvae of *Plutella xylostella* fed on oilseed rape plants treated with jasmonic acid and/or aphid^1^.

Treatment	ECI (%)	ECD (%)	RCR (mg/mg/day)	RGR (mg/mg/day)
Control	4.45 ± 0.18^a^	5.77 ± 0.25^a^	2.83 ± 0.14^a^	0.126 ± 0.006^a^
Aphid	3.99 ± 0.29^a,b^	4.80 ± 0.40^a,b^	2.64 ± 0.18^a^	0.104 ± 0.006^a,b^
JA	3.49 ± 0.14^b^	4.30 ± 0.15^b^	1.88 ± 0.05^b^	0.066 ± 0.003^c^
JA + Aphid	3.83 ± 0.25^a,b^	5.18 ± 0.38^a,b^	2.38 ± 0.11^a,b^	0.091 ± 0.008^b^
df	3, 16	3, 16	3, 16	3, 16
*F*	3.26	3.85	9.97	17.29
*P*	0.049	0.030	0.0006	<0.0001

*^1^The plants were treated with jasmonic acid (5 mM for 2 days) and/or aphid (20 adults for 2 days) prior to determining nutritional indices of P. xylostella. This experiment was replicated seven times. Mean values followed by different letters in the same column are significantly different (Tukey’s test, P < 0.05). ECI, efficiency of conversion of ingested food; ECD, efficiency of conversion of digested food; RCR, relative consumption rate; RGR, relative growth rate; JA, jasmonic acid.*

### Glucose Oxidase Activity

**FIGURE 3:**
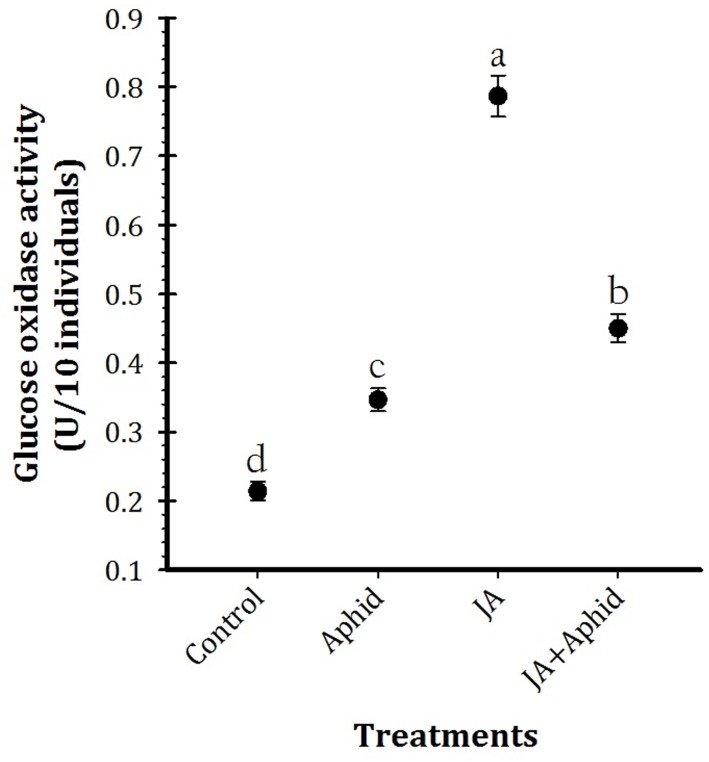
Mean (±SE) glucose oxidase activity of the whole body extracts from *Plutella xylostella* fed on oilseed rape plants treated with jasmonic acid and/or aphid. Each point is average of five replications. Mean values followed by different letters are significantly different (Tukey’s test, *P* < 0.05).

Glucose oxidase activity of salivary gland of *P. xylostella* larvae fed on control and treated oilseed rape plants is presented in **Figure 3** (*F*_3,16_ = 35.98, *P* < 0.01). Quantitative estimation of GOX activity per individual revealed significantly higher enzyme activity in the salivary gland extracts of larvae fed on JA treatment (0.77 U 10 individuals^−1^). In contrast, control plants fed larvae (0.21 U 10 individuals^−1^) possessed the lowest level of GOX activity.

### Trypsin Inhibitor Activity of Control and Treated Plants

**FIGURE 4:**
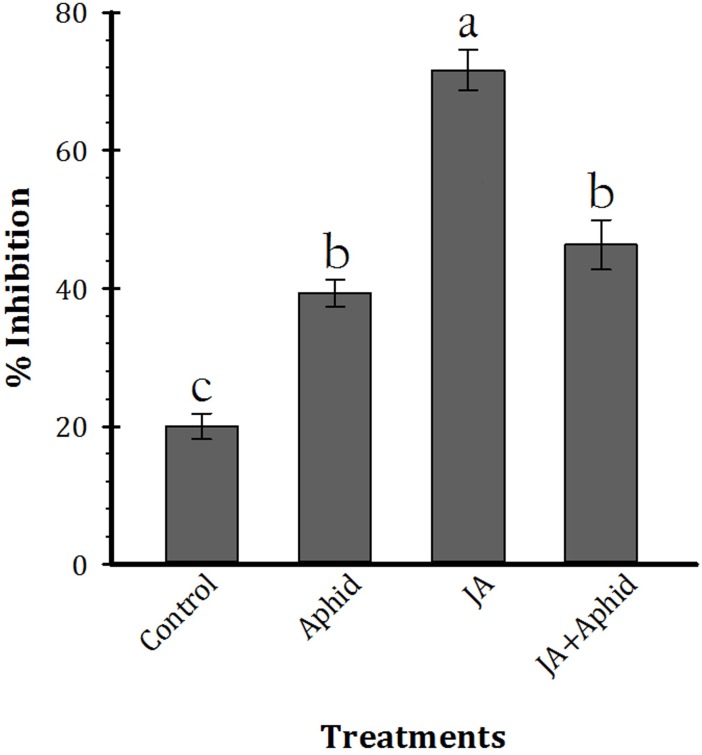
Mean (±SE) percentage inhibition of trypsin activity by leaf extract inhibitors of oilseed rape plants treated with jasmonic acid and/or aphid. Each point is average of five replications. Mean values followed by different letters are significantly different (Tukey’s test, *P* < 0.05).

The effect of leaf extract inhibitors from control and treated plants on trypsin activity is shown in **Figure 4** (*F*_3,16_ = 63.21, *P* < 0.01). The inhibitors of control plants and JA, Aphid, JA + Aphid treatments inhibited 20.0, 39.3, 46.3, and 71.6% of enzyme activity, respectively.

### Glucosinolate Contents

**FIGURE 5:**
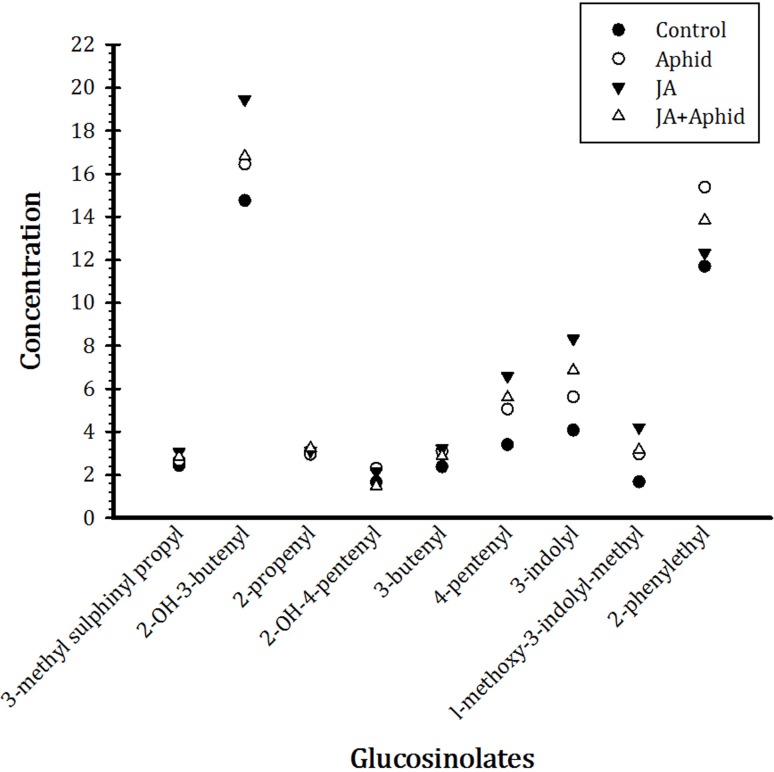
Effect of applying jasmonic acid and/or herbivory on the concentrations of glucosinolates in leaves of oilseed rape plants.

Nine main glucosinolates were detected in treated and control *B. napus* plants belonging to the three chemical classes: six aliphatics (3-Methyl sulfinyl propyl, 2-OH-3-butenyl, 2-propenyl, 2-OH-4-pentenyl, 3-Butenyl, and 4-pentenyl), two indoles (3-indolyl, 1-Methoxy-3-indolylmethyl), and one aromatic (2-Phenylethyl). Total glucosinolate concentration was higher in the treated plants compared to control. In response to application of JA, there were more increases in the concentrations of 2-OH-3-butenyl, 4-pentenyl, and 3-indolyl, being 19.4, 6.6, and 8.3 μmol g^−1^ dw, respectively. Treatment with Aphid resulted in more accumulation of 2-phenylethyl, being 12.3 μmol g^−1^ dw, in plants. The concentrations of other glucosinolates were also increased by treatments, but to a smaller extent (**Figure 5**).

### Correlation Analysis

The analysis of correlation coefficients of the examined biological and physiological characteristics of *P. xylostella* fed on control and treated plants with total glucosinolate content and inhibition of trypsin is shown in **Table 4**. The results of this study revealed that high correlations existed between *r* and FI on one side and total glucosinolate content and inhibition of trypsin on the other. Very high negative correlations were also found between RCR (*r* = 0.982) and RGR (*r* = 0.996) with inhibition of trypsin activity.

**Table 4:** Correlation coefficients (*r*) of some biological and physiological characteristics of *Plutella xylostella* fed on oilseed rape plants treated with jasmonic acid and/or aphid^1^ with total glucosinolate content and inhibition of trypsin.

Parameter	Total glucosinolate		Inhibition of trypsin	
	*r*	*P* value	*r*	*P* value
Survivorship	−0.982	0.009	−0.968	0.016
*r*	−0.916	0.042	−0.916	0.042
FI	−0.916	0.042	−0.881	0.059
RCR	−0.873	0.063	−0.982	0.009
RGR	−0.974	0.026	−0.996	0.002

*^1^The plants were treated with jasmonic acid (5 mM for 2 days) and/or aphid (20 adults for 2 days) prior to determining nutritional indices of P. xylostella. FI, fitness index; RCR, relative consumption rate; RGR, relative growth rate.*

## Discussion

Resistance induced in many plant species is known to influence the fitness and performance of insect pests (Mithöfer and Boland, 2012 and references therein). However, the induced resistance by elicitors did not produce any phytotoxicity or negative effect on natural enemies (Bruce, 2014). Our results showed that both JA and Aphid treatments, as resistance inducers in *B. napus*, had detrimental effects upon *P. xylostella* growth and development, and JA proved to be the most detrimental to this insect pest at the concentration tested. Of course, we did not found positive interactions in the reduction survivorship and life table parameters of *P. xylostella* that was fed on the plants treated with JA + Aphid; probably because of interference in signaling events related to induce resistance by these treatments (Pontoppidan et al., 2003; Cipollini et al., 2004; Ehlting et al., 2008; Zhang et al., 2013). It is reported that inducible defenses in plants against insect herbivores can be strongly influenced by the mix of signals generated by external biotic and abiotic factors (Bostock, 1999; Kranthi et al., 2003; Lambdon and Hassall, 2005). The interactions among signaling cascades triggered by two or several inducers could be synergies and/or antagonisms (Zarate et al., 2007; Koornneef and Pieterse, 2008).

In our study, artificial induction of defense in oilseed rape plants reduced the survivorship of immature stages of *P. xylostella*. Probably, the reduction in the survival rate of *P. xylostella* was because of the increase of inhibitor level in treated plants that affect the feeding, growth, and survival of the immature (Dastranj et al., 2017). Also, inducers are responsible for producing certain secondary metabolites which decrease the survival of herbivores (Thaler et al., 2001). This was also evidenced in *Spodoptera exigua* (Hübner) (Lep.: Noctuidae), feeding on induced foliage (Stout and Duffey, 1996). Correlation coefficients are given in **Table 4** clearly indicate that glucosinolates and trypsin inhibitors have reduced survival of *P. xylostella*.

The slowest population development of the pest was observed on JA treatment; mainly due to the longer development time, the higher morality of immature stages, low fecundity, and a later peak in reproduction. The *r* value was moderate for insect fed on Aphid-treated plants when compared to control and JA treatment. HPLC analysis of plant extracts revealed that total glucosinolate level was 50% higher in JA-treated plants compared to control. It is highly probable that *P. xylostella* performance is most likely driven directly by glucosinolate level which in turn was significantly influenced by the type of treatments (**Figure 5**). The correlation coefficient between *r* and total glucosinolate level support this viewpoint (**Table 4**). Similarly, Bartlet et al. (1999) reported that an increase in the concentration of indole glucosinolates are being induced by the JA treatment and having negative effects on subsequent herbivory. Also, Mouttet et al. (2013) showed that previous feeding by whiteflies negatively affected the performance of leaf miners in locally damaged plants.

Careful examination of the signaling events related to systemic induced resistance has revealed that chemical cascades involving JA are involved in many induced responses against herbivore attack (War et al., 2012). Our results are in compliance with previous studies on chewing pests in this regards. For example, on tomato, foliar JA application significantly inhibited the damage of some leaf feeding caterpillars and flea beetles (Thaler, 1999). Besides glucosinolate, other secondary metabolites and digestive enzyme inhibitors might have changed and influenced the *r* of *P. xylostella*; as its correlation is shown in **Table 4**. Dastranj et al. (2017) reported that plant inhibitors reduced survival and delayed growth and development of the *P. xylostella* larvae.

The GI are considered to be the most important determining factors of immature performance that show whether a host is suitable or unsuitable for feeding insect (Setamou et al., 1999). We found that when *P. xylostella* larvae fed on high-quality host their survival rate increased (**Figure 1**) and completed developmental time faster (data not shown) compared to those insects that fed on a low-quality host. In the present study, it was found that the GI, SII, and FI of *P. xylostella* on control plants were nearly twofold to threefold higher than those on treated plants. According to the correlation analysis, there was a significant negative correlation between the FI of lepidopteran pest and the levels of glucosinolate of plants (**Table 4**). Therefore, the observed effects of individual and combined applications of JA and Aphid on *P. xylostella* GI are likely to be due to these anti-nutritive and/or toxic compounds (Zhang et al., 1992). Our results agreed with those achieved by Tan et al. (2012), who reported exogenous MeJA can elevate the activity of defensive enzymes, like PIs, and reduce the growth rate of *H. armigera*

There was a significant reduction in ECI and ECD, a significant decrease in RCR, and generally lower RGR for *P. xylostella* fed on JA-treated plants compared to control plants. The results of inhibitory assay showed that the higher impairment of the digestive process of trypsin occurs after incubation with the extracts of treated plants (**Figure 4**). Inhibition of digestive enzymes by proteinaceous inhibitors results in micronutrient deficiencies that negatively affect the food intake of the herbivore (Borzoui et al., 2017), which was also proven in our experiment. Also, glucosinolates may target digestive process of *P. xylostella* (Li et al., 2000). Our results are in agreement with previous findings that the treatment of plants by inducers had disruptive effects on feeding performance of herbivores (Lin and Kogan, 1990; Gill et al., 2003; Rayapuram and Baldwin, 2006).

Our results are an additional example of induced changes in the chemical composition and inhibitors level of plants by different treatments. Bartlet et al. (1999) reported wound-induced increases in the glucosinolate content of oilseed rape and their effect on subsequent herbivory by *Psylliodes chrysocephala* (L.) (Coleoptera: Chrysomelidae). Tan et al. (2012) showed that jasmonate induces trypsin inhibitors in tomato leaves. Lu et al. (2014) observed a contrasting effect of ethylene biosynthesis on induced plant resistance against the striped stem borer and the brown planthopper in rice.

In the present study, where the GOX was assayed, we concluded that saliva of *P. xylostella* may play an important role in counteracting the larvae with the induction of secondary metabolites and probably PIs. *P. xylostella* that fed upon treated plants elevated the saliva GOX activity in comparison to those on control plants. Hu et al. (2008) reported that sugars and secondary metabolites are the possible causes of induction of GOX activity. Furthermore, Musser et al. (2002) found that glucose oxidase was the principal salivary enzyme responsible for suppressing the induction of nicotine in wounded tobacco plants.

## Conclusion

Our study has shown that JA and/or prior herbivory by the mealy cabbage aphid could be useful elicitors to elevate oilseed rape plant’s defense against *P. xylostella*. The induction by these elicitors has significantly decreased the life table parameters, GI and nutritional indices of this insect pest. The reason for these effects could be the induction of glucosinolates and PIs following treatment of plants. Also, we found that JA-treated plants were the most preferred host for oviposition by *P. xylostella*; therefore, these plants can be used as trap crops that lure *P. xylostella* adults away from the main crop by providing an alternative site for oviposition. It is hoped that these findings could contribute to a better utilization of inducer-dependent defenses into integrated pest management *P. xylostella*. However, further studies are needed to understand the ecological role of induced defenses in plant interactions with herbivores and their natural enemies.

## Author Contributions

GN-G and EB conceived and designed the research. EB and MS conducted the experiments and analyzed the data. GN-G, EB, MS, and AN contributed analytical tools and wrote the manuscript.

## Conflict of Interest Statement

The authors declare that the research was conducted in the absence of any commercial or financial relationships that could be construed as a potential conflict of interest.
